# Supramolecular nanomaterials based on hollow mesoporous drug carriers and macrocycle-capped CuS nanogates for synergistic chemo-photothermal therapy

**DOI:** 10.7150/thno.40066

**Published:** 2020-01-01

**Authors:** Jie Yang, Dihua Dai, Xinyue Lou, Lianjun Ma, Bailiang Wang, Ying-Wei Yang

**Affiliations:** 1 International Joint Research Laboratory of Nano-Micro Architecture Chemistry, College of Chemistry, and Department of Endoscopics, China-Japan Union Hospital of Jilin University, Jilin University, Changchun 130012, P. R. China; 2 The State Key Laboratory of Refractories and Metallurgy, School of Chemistry and Chemical Engineering, Wuhan University of Science and Technology, Wuhan 430081, P. R. China; 3 School of Ophthalmology & Optometry, Eye Hospital, Wenzhou Medical University, Wenzhou, 325027, P. R. China

**Keywords:** drug delivery, hybrid materials, nanotheranostics, pillararenes, supramolecular chemotherapy

## Abstract

Multifunctional supramolecular nanoplatforms that integrate the advantages of different therapeutic techniques can trigger multimodal synergistic treatment of tumors, thus representing an emerging powerful tool for cancer therapeutics.

**Methods**: In this work, we design and fabricate a multifunctional supramolecular drug delivery platform, namely Fa-mPEG@CP5-CuS@HMSN-Py nanoparticles (FaPCH NPs), consisting of a pyridinium (Py)-modified hollow mesoporous silica nanoparticles-based drug reservoir (HMSN-Py) with high loading capacity, a layer of NIR-operable carboxylatopillar[5]arene (CP5)-functionalized CuS nanoparticles (CP5-CuS) on the surface of HMSN-Py connected through supramolecular host-guest interactions between CP5 rings and Py stalks, and another layer of folic acid (Fa)-conjugated polyethylene glycol (Fa-PEG) antennas by electrostatic interactions capable of active targeting at tumor lesions, in a controlled, highly integrated fashion for synergistic chemo-photothermal therapy.

**Results**: Fa-mPEG antennas endowed the enhanced active targeting effect toward cancer cells, and CP5-CuS served as not only a quadruple-stimuli responsive nanogate for controllable drug release but also a special agent for NIR-guided photothermal therapy. Meanwhile, anticancer drug doxorubicin (DOX) could be released from the HMSN-Py reservoirs under tumor microenvironments for chemotherapy, thus realizing multimodal synergistic therapeutics. Such a supramolecular drug delivery platform showed effective synergistic chemo-photothermal therapy both *in vitro* and *in vivo*.

**Conclusion**: This novel supramolecular nanoplatform possesses great potential in controlled drug delivery and tumor cellular internalization for synergistic chemo-photothermal therapy, providing a promising approach for multimodal synergistic cancer treatment.

## 1. Introduction

Nowadays, chemotherapy is still the mainstay approach for antitumor treatment 1,2, but its undesired side effects 3, limited selectivity 4, as well as the limitations of monotherapy have prompted the emergence of supramolecular chemotherapy and multimodal synergistic cancer therapy 5-7. Photothermal therapy (PTT), as a promising complementary treatment method that can convert NIR energy into hyperthermia, has attracted much attention due to its advantages of high tissue transparency, noninvasive manipulation, and less damage to normal tissues 8-12. Upon combining PTT with supramolecular chemotherapy, multimodal synergistic tumor treatment can be achieved with enhanced therapeutic efficacy, mutually reinforcing superadditive properties, and reduced side effects 13-15.

As a new generation of supramolecular macrocycles, pillar[n]arenes (pillararenes or pillarenes for short) have emerged as a powerful class of tools in biomedicine and nanotheranostics due to its many advantages and unique features 16-25. Recently, in collaboration with Yu and others, we successfully functionalized CuS nanoparticles (NPs), a widely used highly efficient photothermal agent 26-28, with a layer of carboxylatopillar[5]arene (CP5) macrocycles in aqueous solution via a one-pot supramolecular capping approach, and constructed a biocompatible supramolecular nanosystem, exhibiting an enhanced *in vitro* and *in vivo* therapeutic effect.^26^ However, this system suffers from some drawbacks including relatively low drug loading efficiency, lack of multi-stimuli responsiveness, and low integration. On the other hand, due to the fascinating properties of high stability 29-31, large loading capacity 32,33, facile functionalization 34-35, and good biocompatibility 36,37, hollow mesoporous silica NPs (HMSNs) have become suitable nanocarriers for enhancing the accumulation at tumor sites via enhanced permeability and retention effect (EPR effect) and improving the therapeutic effect 38-40. We envision, via the marriage of the abovementioned two systems with supramolecular host-guest chemistry as a magpie bridge, new multifunctional supramolecular drug delivery platform comprising efficient PTT entity, anticancer drug carriers with high loading capacity, and multi-stimuli responsive gating functions 41-47 can be constructed to solve the problems and greatly improve the synergistic therapeutic effects with combined chemotherapy and PTT 48-50. Meanwhile, an active targeting strategy by installing folic acid (Fa) ligand 51 in the supramolecular platform as antennas to recognize the over-expressed folate receptor (FR) on tumor cells can also be integrated in the design to improve cell internalization via the route of folate-mediated endocytosis 52,53.

**Figure 1:**
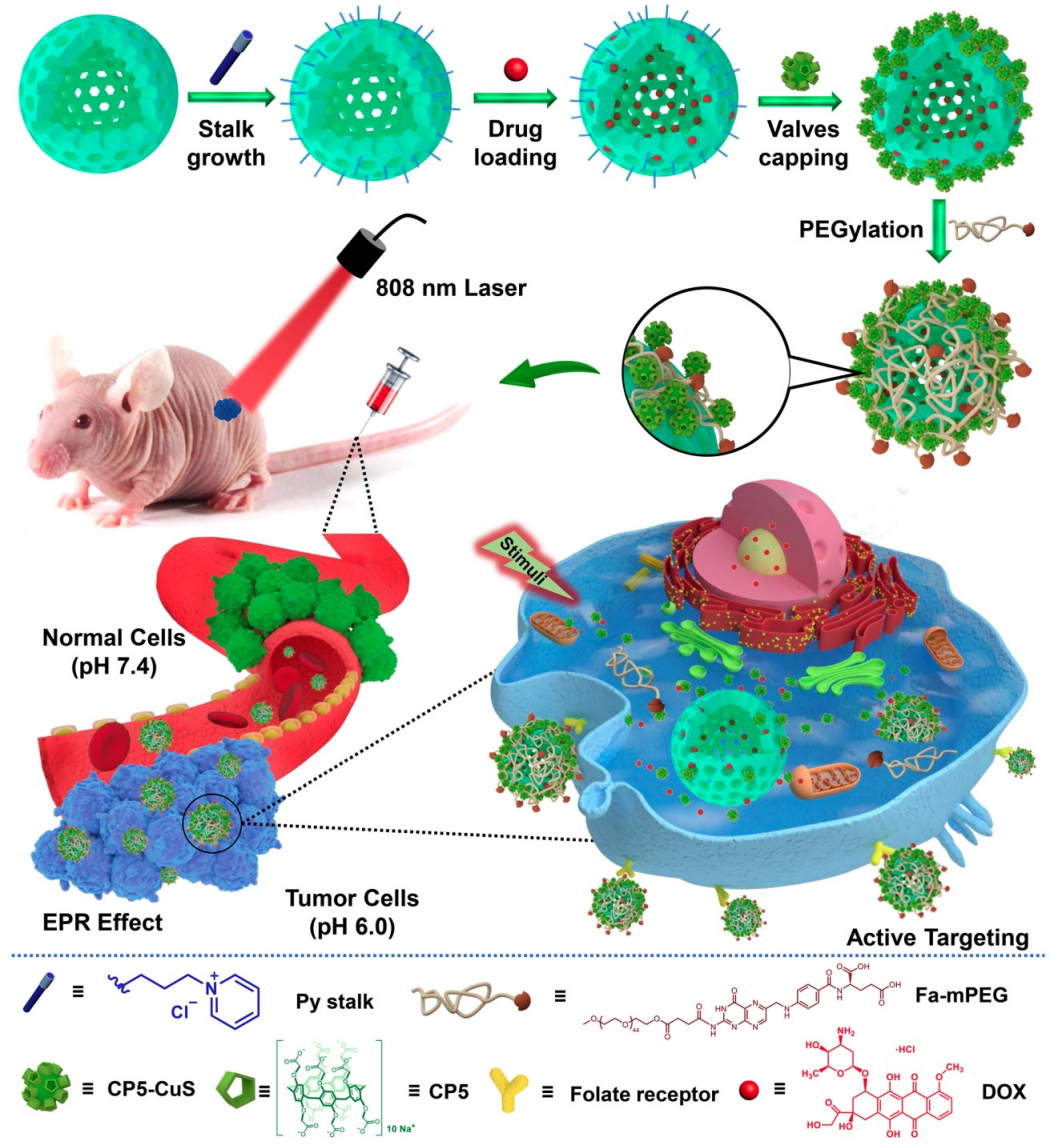
Schematic diagram for the preparation of the multifunctional supramolecular nanomaterial and its application in synergistic chemo-photothermal therapy and the legends of the representative components in the supramolecular nanosystem.

Inspired by the abovementioned key points, herein a multifunctional supramolecular nanomaterial, namely Fa-mPEG@CP5-CuS@HMSN-Py NPs (FaPCH NPs), has been constructed by installing CP5-functionalized ultrasmall CuS NPs (CP5-CuS) as both gating entities and photothermal agents on pyridinium (Py)-modified HMSNs (HMSN-Py) via the host-guest complexation of CP5 rings and Py stalks, followed by further surface-coating with Fa-modified polyethylene glycol (Fa-mPEG) as active targeting antennas via electrostatic interactions (**Figure 1**). In this integrated system, (a) HMSN-Py ensures efficient loading of anticancer drug doxorubicin (DOX), (b) CP5-CuS nanovalves not only guarantee the controllable drug release under quadruple stimuli including temperature, pH, competitive binding, and NIR light and reduce the side effects caused by drug burst release, but also achieve photothermal conversion efficiency under 808 nm laser irradiation, serving as photothermal agents, (c) PEGylation of Fa-mPEG endows the hybrid nanosystem with enhanced biocompatibility and active targeting, improving cellular internalization of tumor cells. Both in vitro and *in vivo* experimental results further demonstrate that the prepared supramolecular drug delivery platform possesses superior capacity of tumor inhibition through synergistic chemo-photothermal therapy.

## 2. Results and Discussion

### 2.1 Preparation and Characterization of FaPCH NPs

**Figure 2:**
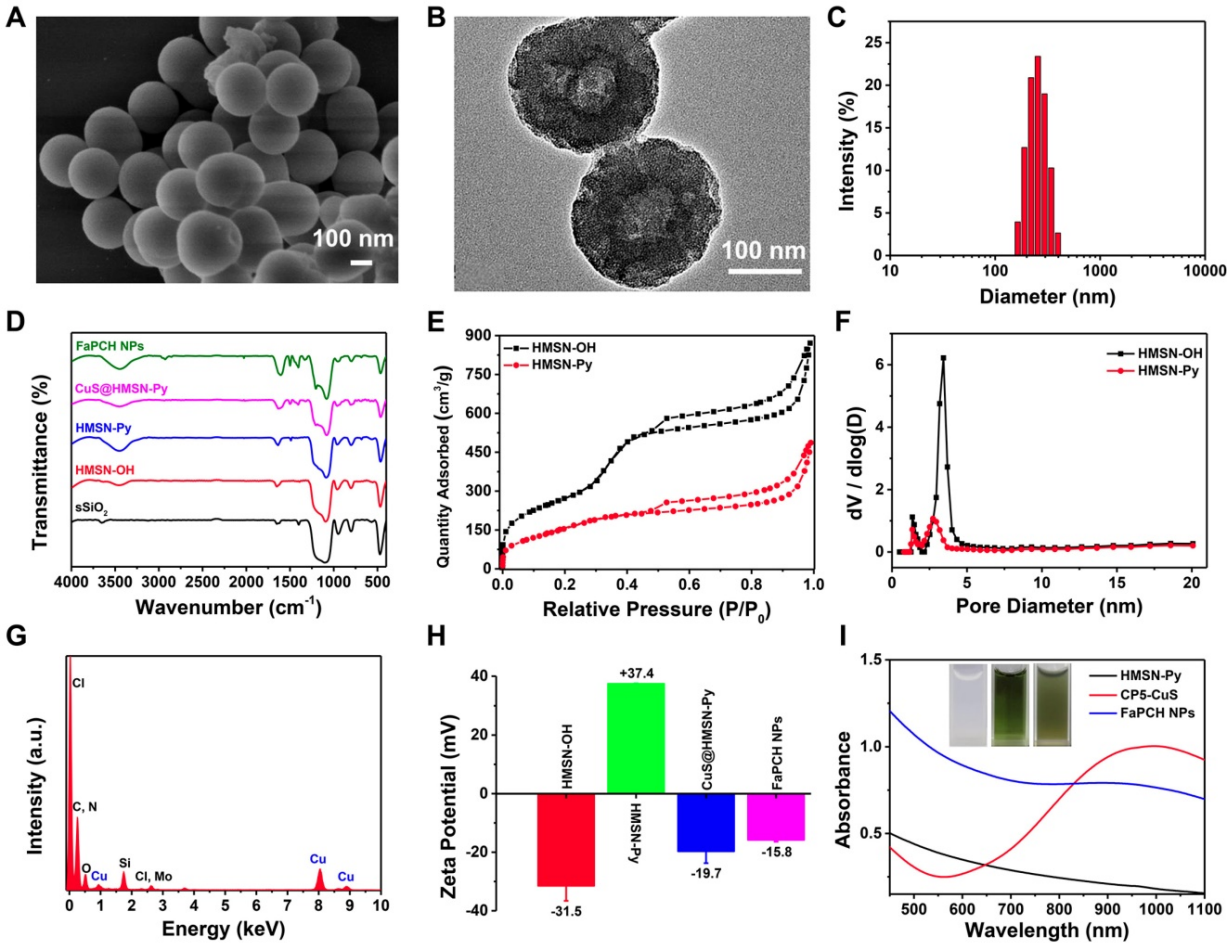
(A) SEM image and (B) TEM image of HMSN-Py NPs. (C) Average hydrodynamic diameter distribution of HMSN-Py NPs. (D) FT-IR spectra of sSiO_2_, HMSN-OH, HMSN-Py, CuS@HMSN-Py and FaPCH NPs. (E) Nitrogen adsorption-desorption isotherms and (F) pore size distributions of HMSN-OH and HMSN-Py. (G) Energy dispersive spectroscopy (EDS) of CuS@HMSN-Py. (H) Zeta potential values of HMSN-OH, HMSN-Py, CuS@HMSN-Py and FaPCH NPs. (I) UV-vis-NIR absorption spectra and photographs (inset) of HMSN-Py, CP5-CuS, and FaPCH NPs dispersions.

In this study, the nonporous solid core of sSiO_2_ was synthesized via a conventional method and the shell of mesoporous silica was homogeneously coated on the surface of sSiO_2_ in alkaline solution with cetyltrimethylammonium bromide (CTAB) as the mesoporous template and tetraethylorthosilicate (TEOS) as the silica source. After elimination of sSiO_2_ and CTAB, the surface of the obtained HMSN-OH was post-modified with a stalk component terminated with Py to afford Py-modified hollow mesoporous silica nanoparticles (HMSN-Py). Scanning electron microscopy (SEM) and transmission electron microscopy (TEM) images show a spherical structure of sSiO_2_ with an average diameter of 60 nm. The average hydrodynamic diameter by dynamic light scattering (DLS) measurement is 68.1 nm, in consistency with the results of SEM and TEM (**Figure S1**). HMSN-Py was also characterized by SEM (**Figure 2A and S2A**), TEM (**Figure 2B and S2B**), and DLS (**Figure 2C**), suggesting an average diameter of 250 nm. Furthermore, the TEM image shows that HMSN-Py has a hollow structure and the thickness of the mesoporous shell is ca. 85 nm (**Figure 2B**). Fourier transform infrared (FT-IR) spectroscopy demonstrated the successful modification of Py on HMSN-OH. The characteristic peak at 3450 cm^-1^ belongs to the -OH of HMSN-OH, and the appearance of characteristic peaks of 1488 cm^-1^ was assigned to the stretching vibration of the benzene ring, directly revealing the successful modification of Py on HMSN-OH (**Figure 2D**). The powder X-Ray diffraction (PXRD) patterns showed that the HMSN-Py still possesses characteristic diffraction peaks corresponding to HMSN-OH after modification of the Py, suggesting the existence of ordered 2D hexagonal pores (**Figure S3A**). As seen from the thermogravimetric analysis (TGA) curves of HMSN-OH and HMSN-Py, the weight underwent a decrease below 100 °C, which could be attributed to the physical adsorption of water in both HMSN-OH and HMSN-Py materials. The TGA curve of HMSN-Py shows that the weight loss of the Py is ca. 18.4% between 220 °C and 400 °C (**Figure S3B**). N_2_ adsorption-desorption isotherms reveal that both HMSN-OH and HMSN-Py are type IV curves. Besides, the Brunauer-Emmett-Teller (BET) surface area and pore diameters of HMSN-OH were determined to be 998.18 m^2^/g and 1.3 nm and 3.4 nm, and after modification with Py, the BET surface area of the material was decreased to 595.22 m^2^/g and the pore diameters were 1.3 nm and 2.7 nm, indicating great drug loading capacity of the carrier (**Figure 2E and F**).

Although the large pore diameter of HMSN-Py NPs facilitated the loading capacity of drug molecules, the leakage of DOX during preparation and the excessive drug burst release have to be taken into account. To overcome these problems, ultrasmall CuS NPs equipped with CP5 macrocycles (CP5-CuS) were synthesized and used as gating entities on HMSN-Py to achieve controlled release of DOX under physiological pH condition. The TEM image of ultrasmall CP5-CuS (**Figure S4**) shows that its average diameter is 6.2 nm. The binding energies of Cu 2p_3/2_ and Cu 2p_1/2_ at 932 and 952 eV were distributed to Cu (I), and the small peaks at 933.9 and 955.2 were assigned to the presence of Cu (II). The spectrum showed the peaks of S 2p at 161.7 and 160.9 eV for S 2p_3/2_ and S 2p_1/2_, respectively (**Figure S5A-C**).

CP5 (**Figure S6**), a water-soluble synthetic macrocycle with an electron-rich cavity, possesses multiple carboxylate groups 54,55 to form coordination bonds on the surface of CuS, endowing CP5-CuS with good host-guest complexation property. Benefiting from these features, CP5-CuS has been immobilized on the surface of HMSN-Py by host-guest interactions between the Py stalks of HMSN-Py and the CP5 rings of CP5-CuS (**Figure S5D**), resulting in the hybrid NPs named CuS@HMSN-Py. Energy dispersive spectroscopy (EDS) of CuS@HMSN-Py further verified the distributions of Si, O, Cu, C, and Cl, intensely illustrating the successful immobilization of CP5-CuS on HMSN-Py (**Figure 2G**). In addition, the high-resolution TEM image and elemental mapping images of FaPCH NPs also suggested that CP5-CuS NPs were distributed on HMSN-Py carrier (**Figure S5E**).

Inspired by PEGylation that can enhance the accumulation of NPs at the target site by EPR effect, and Fa can increase the active targeting of tumor cells via recognition of FR, Fa-mPEG conjugate was synthesized and introduced to the CuS@HMSN-Py system to produce multifunctional supramolecular drug delivery platform FaPCH NPs. FT-IR and UV-vis spectra verified the introduction of PEG-Fa to FaPCH NPs. The characteristic peaks of UV-vis absorption spectra at 360 nm of Fa-mPEG suggested the presence of Fa (**Figure S7A**). The peaks at 1200, 1326, and 1400 cm^-1^ were due to the presence of CP5 in the system. The peak at 2927 cm^-1^ corresponds to the -CH_2_- stretching vibration of mPEG and the variation of peaks at 1488 and 1610 cm^-1^ can be attributed to Fa (**Figure 2D**). In addition, the surface zeta potential values changed from negative (-31.4 mV) to positive (37.3 mV) after the modification of the Py component, yet turned to negative (-19.6 mV) after immobilization of CP5-CuS on the surface, and eventually turned relatively less negative (-15.8 mV) owing to the shielding effect of Fa-mPEG (**Figure 2H**). Meanwhile, the weight content of CP5-CuS in FaPCH NPs was calculated to be 10.9% (**Figure 2I and Figure S7B, C**).

### 2.2 Photothermal Property of FaPCH NPs

**Figure 3:**
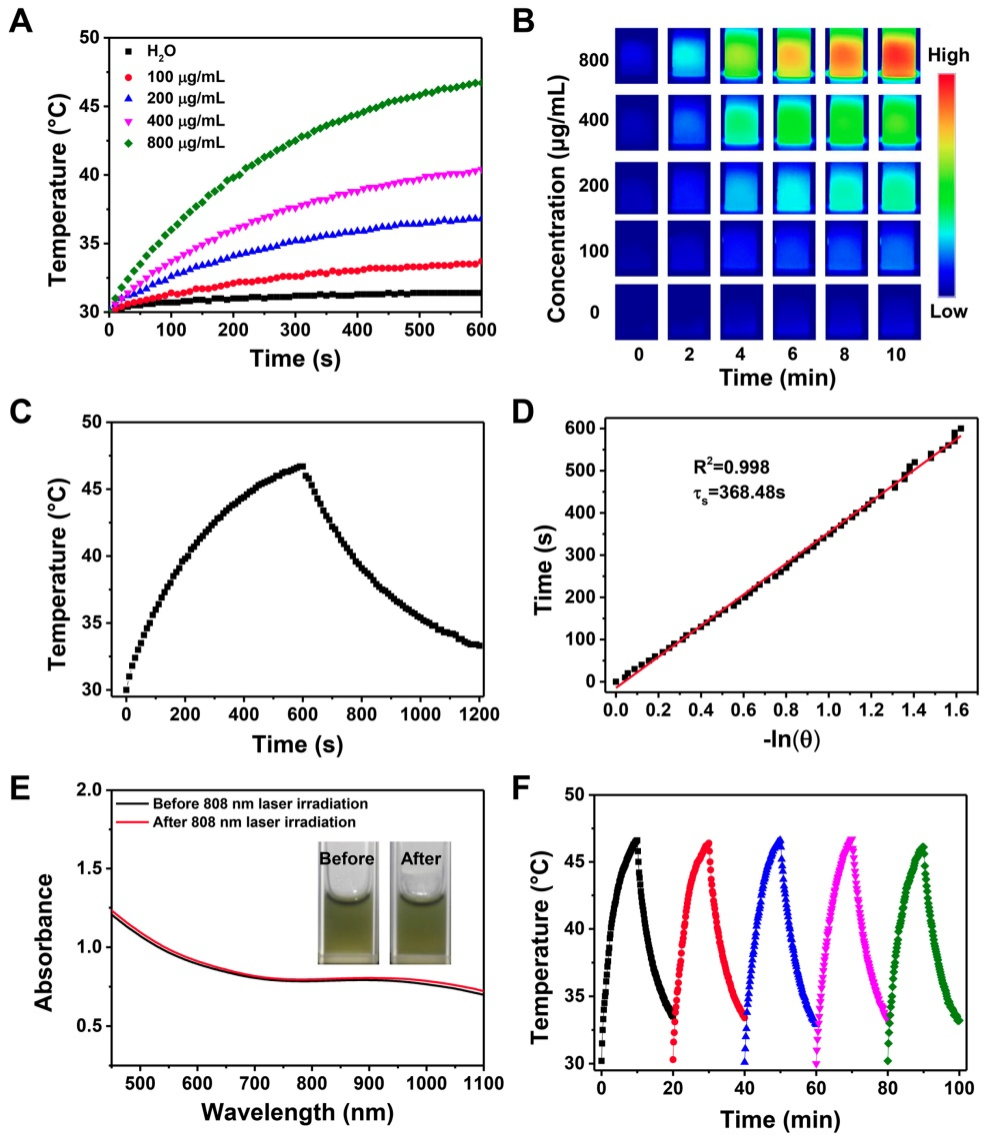
(A) Temperature changes and (B) infrared thermal images of various concentrations of FaPCH NPs dispersions under 808 nm laser irradiation with the power density of 1 W/cm^2^ for 10 min. (C) Photothermal effect curve of FaPCH NPs dispersion under ON/OFF 808 nm laser irradiation with the power density of 1 W/cm^2^. (D) Plot and linear fitting of cooling time versus -ln(θ) received from cooling section of photothermal effect curve in (C). (E) UV-vis-NIR absorption spectra of FaPCH NPs before and after 808 nm laser irradiation. Inset: Photographs of FaPCH NPs dispersions before and after 808 nm laser (1 W/cm^2^) irradiation for 10 min. (F) Photothermal effect curve of FaPCH NPs dispersion under 808 nm laser irradiation with the power density of 1 W/cm^2^ for five cycles.

The introduction of CP5-CuS endowed the multifunctional supramolecular nanomaterials with desired PTT property. Compared with HMSN-Py, FaPCH NPs showed a strong absorbance in the NIR region (**Figure 2I**), revealing its great potential in NIR-guided PTT. To verify this, temperature elevation of the FaPCH NPs dispersions with different concentrations in aqueous solutions were recorded attentively under irradiation of various power densities of 808 nm NIR laser for 10 min. The significant temperature increments were positively correlated with the concentration of FaPCH NPs (**Figure 3A and B**) and the power density of laser (**Figure S8**). In sharp contrast, the same amount of water only increased 1.4 °C at the same condition. The photothermal conversion efficiency of the FaPCH NPs was ca. 19% (**Figure 3C and D**) according to the reported method. Moreover, The UV-vis-NIR absorption spectra of FaPCH NPs dispersions showed no obvious difference before and after 808 nm laser (1 W/cm^2^) irradiation for 10 min (**Figure 3E**), and temperature increment of FaPCH NPs performed no decrease after 5 cycles of 808 nm laser irradiation, verifying the durable photothermal stability of FaPCH NPs (**Figure 3F**). All the aforementioned results suggest the great potential of FaPCH NPs for PTT.

### 2.3 Drug Release Kinetics of DOX-loaded FaPCH NPs (FaPCHD NPs for short)

**Figure 4:**
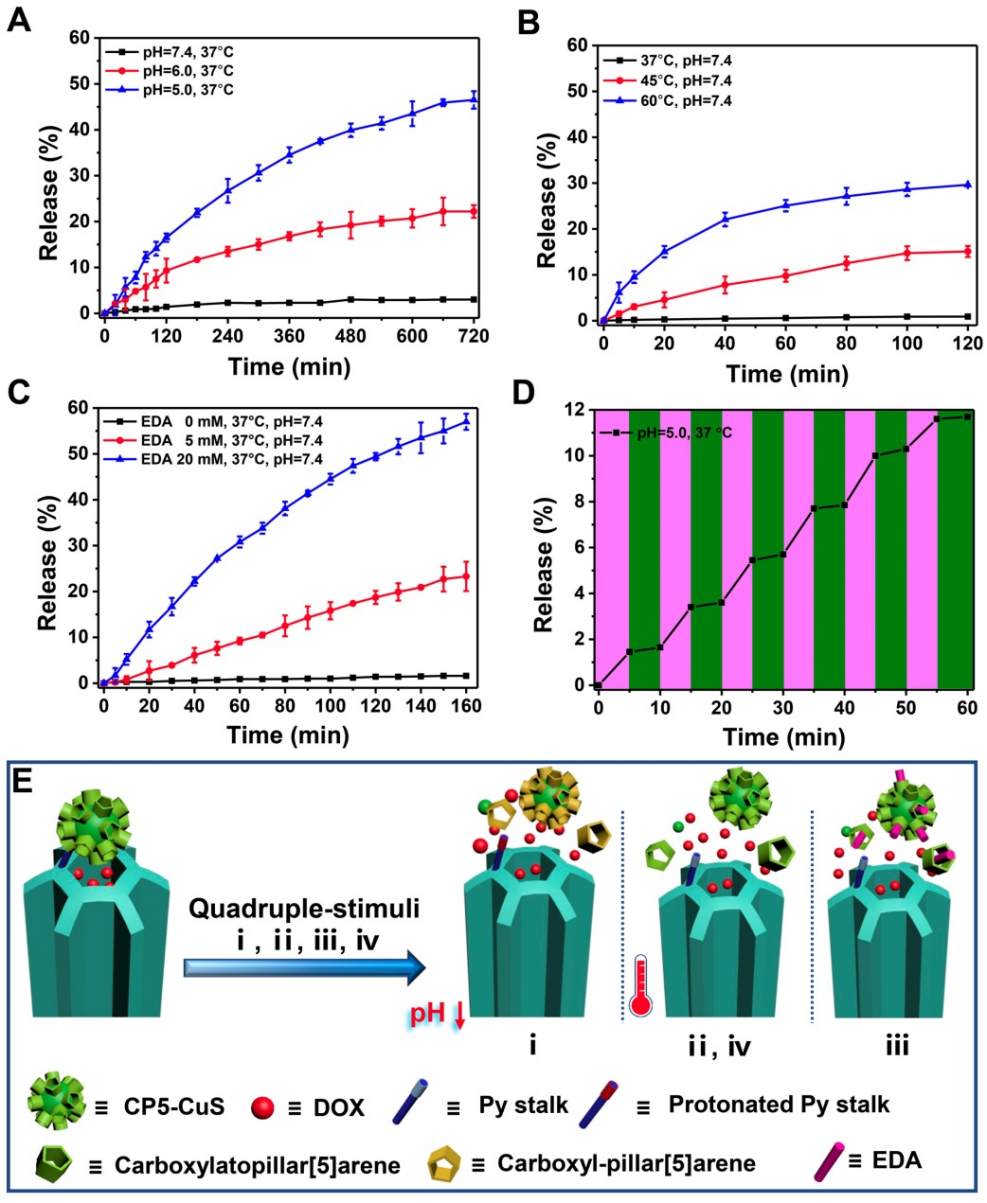
DOX release kinetics from FaPCHD NPs in response to various external stimuli: (A) pH, (B) temperature, (C) ethylenediamine (EDA), and (D) 808 nm NIR laser "pulsed" ON/OFF irradiation with the power density of 1 W/cm^2^. (E) Schematic diagram of nanogates operation on HMSN-Py in response to quadruple-stimuli (i, pH; ii, temperature; iii, competitive agent; iv, NIR light) for controlled DOX release.

Increasing the loading capacity of drug payload, minimizing the premature release of the drug under physiological conditions, and increasing the release amount at the tumor site are beneficial to improving the therapeutic effect and reducing the side effects of therapeutic materials in drug delivery. To prove that CP5-CuS play a significant role in the controlled release of drugs, drug loading capacities of the multifunctional supramolecular drug delivery platform with or without CP5-CuS nanovalves were studied and calculated to be 0.5 g/g and 0.74 g/g, respectively, according to Lambert-Beer law (**Figure S9**). It revealed that the presence of CP5-CuS obviously reduced the loss of loaded drug during post-processing. The release kinetics of DOX from FaPCHD NPs under different stimuli including pH, temperature, ethylenediamine (EDA), and NIR light irradiation were monitored. Under physiological condition (pH 7.4 PBS), DOX release from FaPCHD NPs was negligible. Notably, under pathological conditions (pH 6.0 and 5.0 PBS), as the acidity increased, the release of DOX got faster, yet no serious burst occurred (**Figure 4A**). Besides, raising the temperature also accelerated DOX release (**Figure 4B**). Moreover, highly expressed polyamines in tumor cells were considered as another stimulus to increase drug release at targeted sites. EDA was selected as a model stimulator 56 to investigate DOX release behavior from the FaPCHD NPs (**Figure 4C**). DOX release was increased upon addition of EDA, however, without EDA, the release was negligible. Furthermore, the drug release kinetics under 808 nm laser irradiation with a constant interval were also studied. With the 808 nm laser "pulsed" ON/OFF irradiation, DOX release profile in FaPCHD NPs exhibited a ladder type release kinetics (**Figure 4D**). DOX release behaviors under these conditions confirmed the importance of supramolecular nanovalves in controlled drug delivery and suggested that the as-prepared FaPCHD NPs were capable of controlled release in response to various stimuli via variable host-guest interactions between Py and CP5 (**Figure 4E**). The presence of supramolecular gates of CP5-CuS effectively expanded DOX loading capacity, and reduced side effects and premature release, which unearthed the potential of synergistic chemo-photothermal therapy.

### 2.4 Biocompatibility of FaPCH NPs

**Figure 5:**
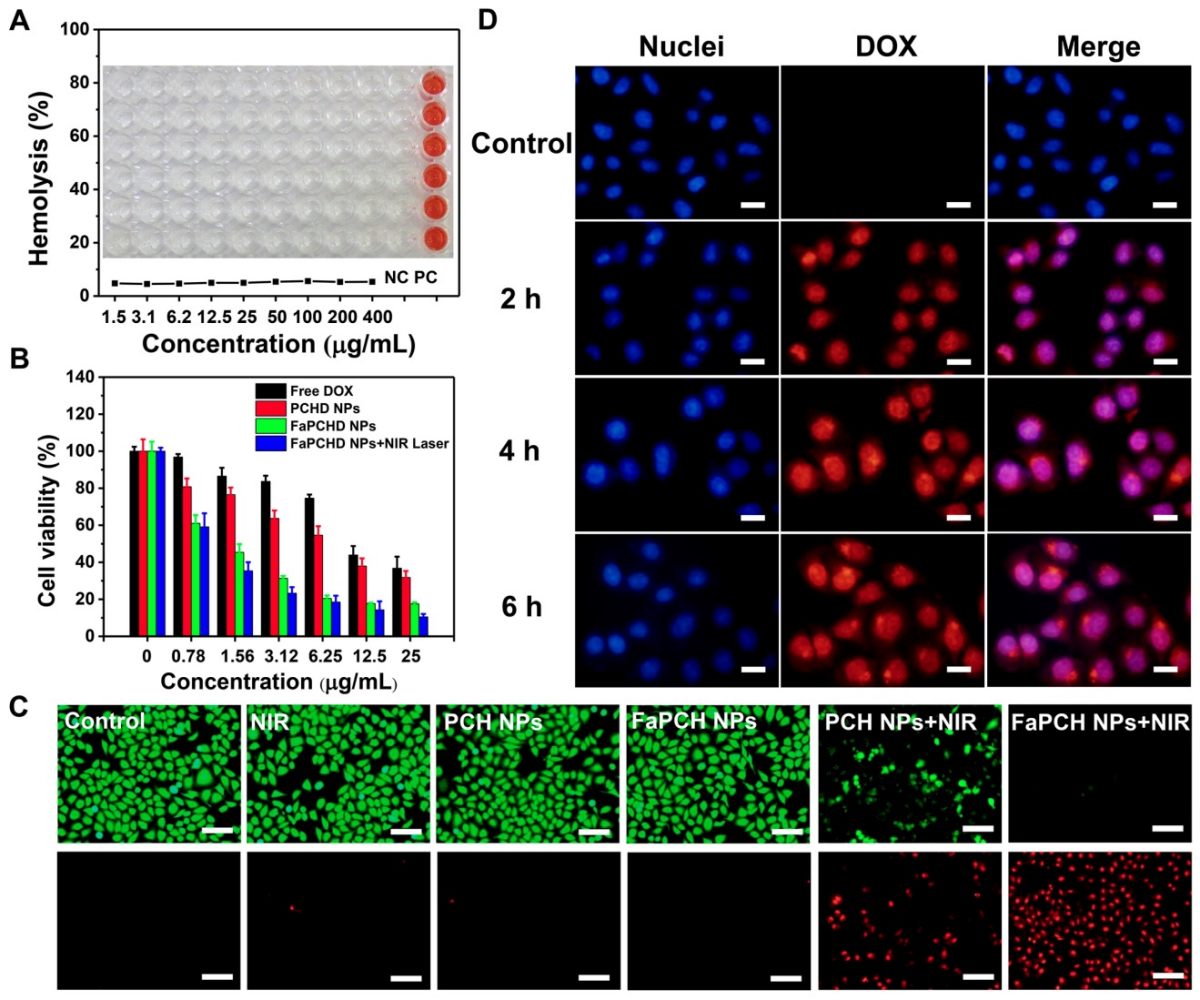
(A) Hemolysis test of various FaPCH NPs (concentration range from 1.5-400 μg/mL; each column of wells in the picture represents six parallel results; NC: control group; and PC: positive control group). Inset picture: the photograph of visualized hemolysis effect. (B) Cell viabilities of HeLa cells incubated with free DOX, PCHD NPs, FaPCHD NPs, and FaPCHD NPs plus 808 nm NIR laser (1 W/cm^2^) for 10 min. (C) Photothermal ablation effect on HeLa cells after different treatments: control, 808 nm laser irradiation (1 W/cm^2^) for 10 min, only PCH NPs, only FaPCH NPs, PCH NPs plus 808 nm laser for 10 min, and FaPCH NPs plus 808 nm laser irradiation for 10 min. Scale bar: 100 μm. (D) Fluorescence images of HeLa cells incubated with FaPCHD NPs for 0 h, 2 h, 4 h and 6 h. The cell nuclei were stained as blue by Hoechst 33342, red was the fluorescence of DOX. Scale bar: 20 μm.

Good biocompatibility is an important factor for the application of therapeutic materials. Cytotoxicity of the as-prepared FaPCH NPs was evaluated by MTT assay on HeLa cells (cancer cell, FR-high expression), A549 cells (cancer cell, FR-low expression), macrophage, and IOSE80 cells (normal cell). All the results showed high cell viability after incubation with FaPCH NPs for 24 h and 48 h (**Figure S10**). To further investigate the biocompatibility of FaPCH NPs, hemolysis effect of FaPCH NPs was evaluated. As shown in **Figure 5A**, the FaPCH NPs had negligible destructiveness to red blood cell (RBC), demonstrating the good biocompatibility of FaPCH NPs and promising application in drug delivery and controlled release.

### 2.5 Photothermal Ablation Effect and Synergistic Chemo-photothermal Therapy

The photothermal ablation effect of FaPCH NPs on HeLa cells was evaluated by calcein-AM/PI staining after 808 nm laser irradiation for 10 min. No obvious fluctuations of the viabilities of HeLa cells occurred after only irradiation with 808 nm laser (1 W/cm^2^) for 10 min or treatment with PCH NPs and FaPCH NPs, indicating the noninvasiveness of 808 nm laser irradiation. In contrast, almost all HeLa cells incubated with FaPCH NPs died under 808 nm laser (1 W/cm^2^) irradiation for 10 min owing to the photothermal ablation effect of FaPCH NPs (**Figure 5C**). Meanwhile, compared with FaPCH NPs plus NIR laser treatment group, only some HeLa cells incubated with PCH NPs died after 808 nm laser irradiation for 10 min, indicating that the modified molecule of Fa in FaPCH NPs increased the HeLa cells uptake ability of FaPCH NPs, and resulting in an enhanced photothermal ablation effect. Subsequently, the synergistic chemo-photothermal therapy ability of FaPCHD NPs was also studied. As in **Figure 5B**, in comparison with free DOX and PCHD NPs treatment groups, cell viability was remarkably lower in FaPCHD NPs treatment group with the same drug concentration as free DOX, owing to the sustained drug release effect of FaPCHD NPs and the active targeting effect of Fa in FaPCHD NPs. Significantly, compared with the above two groups, FaPCHD NPs plus 808 nm NIR laser treatment group showed superior cell killing ability, attributing to the advantage of synergistic supramolecular chemo-photothermal therapy.

### 2.6 Cellular Uptake and Anti-cell Migration Ability of FaPCHD NPs

To visually observe the drug delivery of the multifunctional supramolecular nanomaterials and evaluate the tumor-targeting ability of Fa ligand, fluorescence intensity of DOX in HeLa cells (FR-high expression) and A549 cells (FR-low expression) incubated with FaPCHD NPs was recorded. The red fluorescence of DOX was observed in HeLa cells and A549 cells after incubation with FaPCHD NPs for 2 h, and increased with the proceeding incubation time (**Figure 5D**), which indicated that FaPCHD NPs was internalized into these two cells. The red fluorescence of DOX overlapped with the blue fluorescence of cell nuclei, demonstrating that DOX was released from FaPCHD NPs and entered the cell nuclei, and meanwhile the fluorescence intensity was continuously enhanced with incubation time. However, the fluorescence intensity in A549 cells was weaker than that in HeLa cells after incubation with FaPCHD NPs at the same incubation time (**Figure S11**). To quantify the fluorescence intensity of HeLa cells and A549 cells, flow cytometry analysis was performed. The intracellular fluorescence intensity increased with incubation time (**Figure S12A and B**), in consistency with the fluorescence microscopy results. Moreover, after incubating the cells with FaPCHD NPs for 6 h, the fluorescence intensity in HeLa cells was 1.9 times of that in A549 cells (**Figure S12C and D**), owing to the ligand-receptor recognition between highly expression of FR in HeLa cells and the active targeting group Fa of FaPCHD NPs, promoting the cellular internalization of FaPCHD NPs. Moreover, the active targeting ability of FaPCHD NPs was further verified by preincubating HeLa cells with free Fa. HeLa cells incubated with FaPCHD NPs exhibited stronger red fluorescence than those with FaPCHD NPs plus free Fa (**Figure S13**), because free Fa blocked the recognition of FR and FaPCHD NPs, further inhibiting the FR-mediated endocytosis.

### 2.7 Synergistic Chemo-Photothermal Therapy* In Vivo*

**Figure 6:**
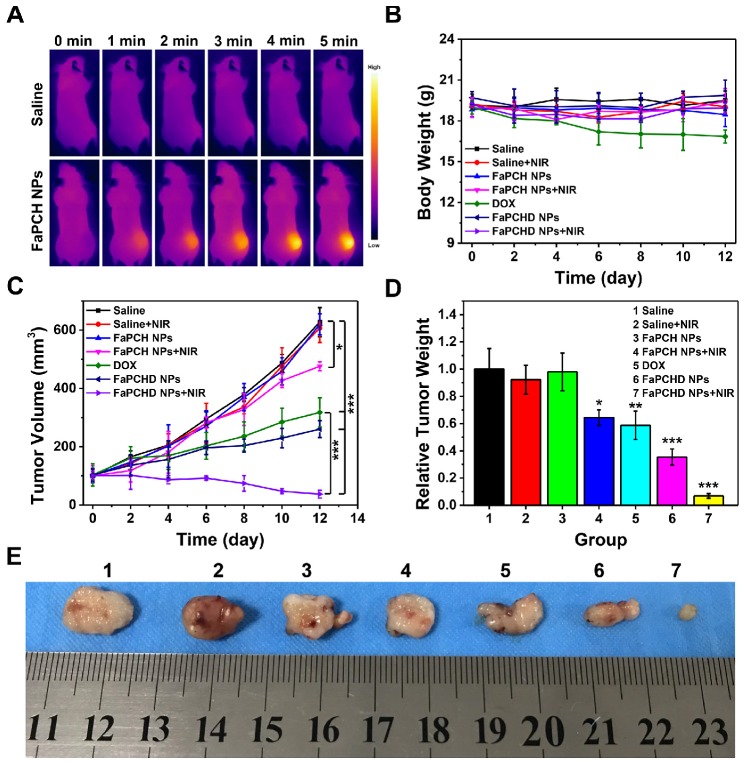
(A) Infrared thermal images of the HeLa tumor-bearing xenograft nude mice after intravenous injection with FaPCH NPs dispersion under 808 nm laser irradiation (1 W/cm^2^) for 5 min. (B) Body weight curves and (C) tumor volume curves of tumor-bearing xenograft nude mice in different treatment groups during treatment. (D) tumor weight and (E) representative photographs of tumor-bearing xenograft nude mice treatment with (1) saline group, (2) saline+NIR group, (3) FaPCH NPs group, (4) FaPCH NPs+NIR group, (5) free DOX group, (6) FaPCHD NPs group, and (7) FaPCHD NPs+NIR group (* p <0.05, ** p < 0.01, and *** p < 0.01).

Inspired by the antitumor effect of FaPCHD NPs *in vitro*, the antitumor efficacy in the HeLa tumor-bearing xenograft nude mice was performed. To visually study the photothermal effect of FaPCH NPs, the temperature changes *in vivo* were obtained via an infrared thermal camera. As shown in **Figure 6A**, the saline group mice showed little change in temperature under 808 nm laser irradiation for 5 min. In contrast, the temperature of tumor site in FaPCH NPs group increased to 48.2 °C via 808 nm laser irradiation for 5 min after intravenous injection with the nanomaterial of FaPCH NPs, indicating a great potential for PTT. Subsequently, the ability of synergistic chemo-photothermal therapy of FaPCHD NPs was evaluated. There was no significant change in body weight of mice during the treatment period except for the DOX treatment group, indicating large side effects of chemotherapy with free DOX in mice and the importance of supramolecular nanomaterials of as-prepared FaPCHD NPs (**Figure 6B**). Meanwhile, the antitumor effect of different treatment groups was studied. As shown in **Figure 6C**, the tumor volume of saline group, saline+NIR group and FaPCH NPs group increased rapidly during the experimental period, the FaPCH NPs+NIR treatment group showed a slight tumor suppressive effect. The therapeutic effect of free DOX group and FaPCHD NPs group were better than those of the FaPCH NPs+NIR group, but lower than that of FaPCHD NPs+NIR group, demonstrating the inhibitory effect of mono-chemotherapy on tumor. Significantly, it can be seen that FaPCHD NPs+NIR group had the best treatment efficiency on tumor, indicating the best therapeutic effect of synergistic chemo-photothermal therapy. The tumor weight and the representative tumor photographs in different treatment groups were shown in **Figure 6D, E**. The saline group, saline+NIR group, and FaPCH NPs group had the largest tumor weight, followed by the FaPCH NPs+NIR group. Free DOX group and FaPCHD NPs group were smaller. Significantly, compared with all the above groups, the FaPCHD NPs+NIR group showed the smallest tumor weight, which was consistent with the results of tumor volume. The results demonstrated the superadditive properties of local hyperthermia caused by NIR laser and chemotherapy.

**Figure 7:**
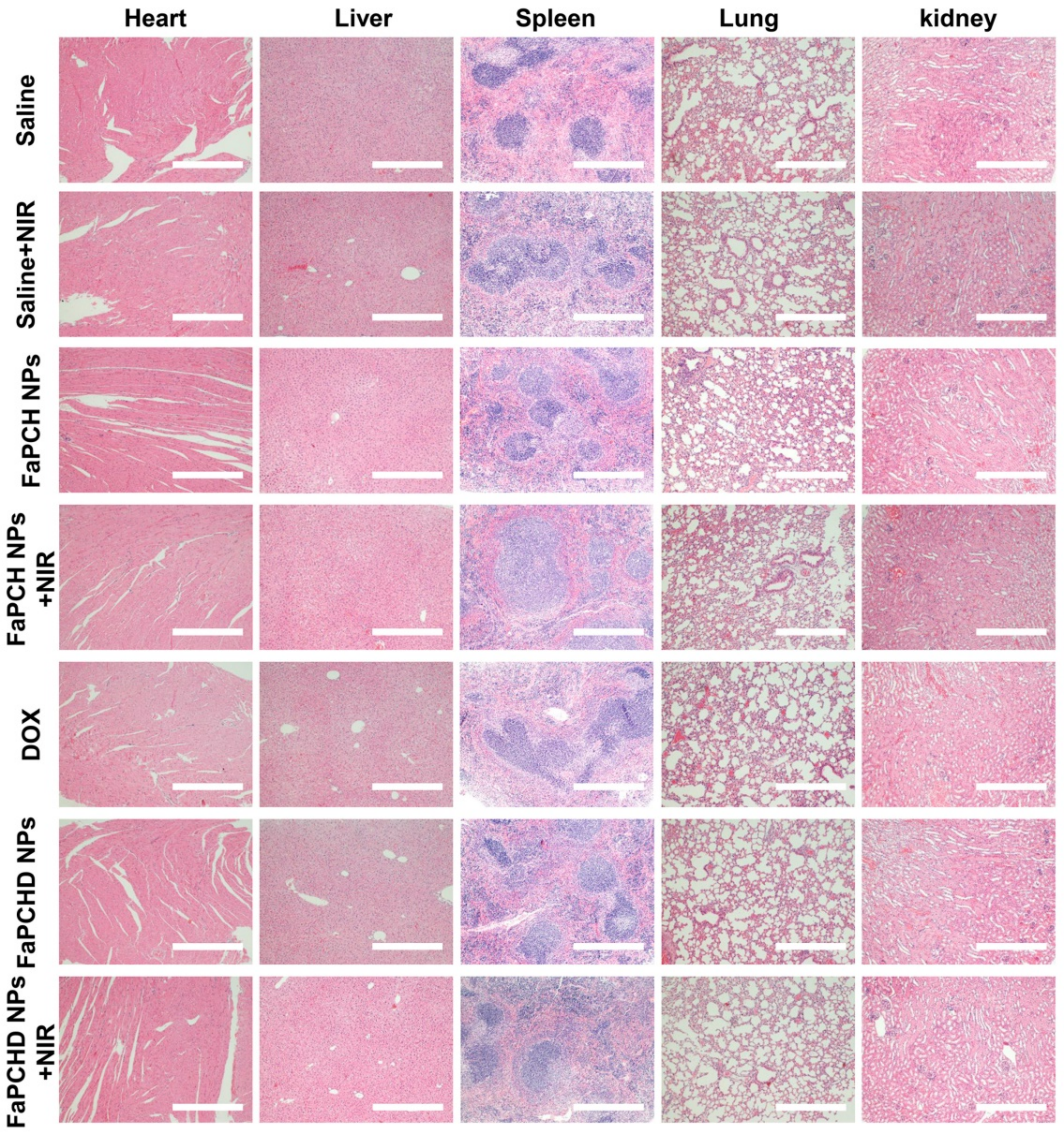
Representative images of H&E staining of heart, liver, spleen, lung and kidneys in different treatment groups. Scale bars: 500 µm.

To assess the potential side effects of FaPCH NPs on the major organs of mice, the heart, liver, spleen, lung, and kidney were harvested and stained with hematoxylin and eosin (H&E) for histological analysis after each treatment. No obvious inflammatory damage and tissue damage in all major organs was observed, indicating that this multifunctional supramolecular nanomaterial can serve as a potential drug delivery platform with good biocompatibility (**Figure 7**).

### 2.8 *In vivo* Biodistribution Study

**Figure 8:**
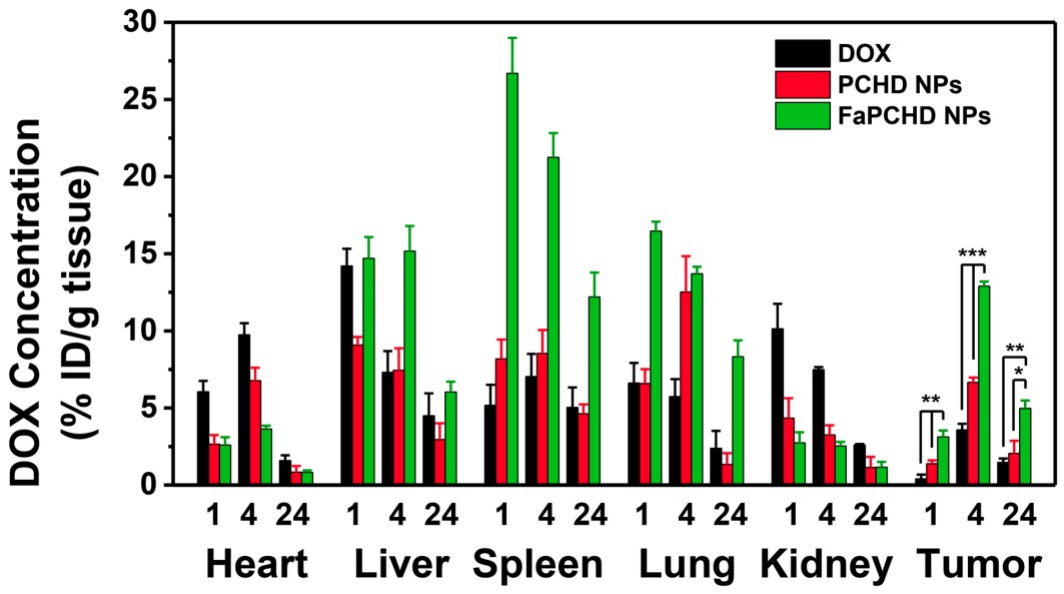
The *in vivo* biodistribution of DOX in HeLa bearing BALB/c nude mice at 1, 4, and 24 h post injection of DOX, PCHD NPs, and FaPCHD NPs (* p <0.05, ** p < 0.01, and *** p < 0.01).

To study the *in vivo* biodistribution of the nanomaterials and the targeting effect of Fa, free DOX, PCHD NPs, and FaPCHD NPs were injected intravenously into HeLa bearing nude mice for 1, 4, and 24 h. Subsequently, the contents of DOX in major organs and tumors were assessed via fluorescence spectroscopy. As shown in **Figure 8**, After intravenous injection of free DOX for 1 h, a large fraction of DOX was detected in the heart, liver, and kidney, and a small amount of DOX accumulated in the tumor site, which could weaken the antitumor effect of DOX and cause severe side effects. Notably, PCHD NPs and FaPCHD NPs treatment groups exhibited a low accumulation of DOX in heart, which was beneficial in reducing the toxicity of DOX to the heart tissue during cancer treatment. Moreover, FaPCHD NPs showed higher accumulation of DOX than that of the free DOX treatment group and PCHD NPs treatment group in tumor site after 1 h of injection. Meanwhile, FaPCHD NPs still had a high DOX accumulation of ca. 12.9% at 4 h and 5% at 24 h in the tumor sites, which was due to the passive targeting by EPR effect and the active targeting via Fa in FaPCHD NPs.

## 3. Conclusion

In summary, we have successfully designed and prepared a multifunctional supramolecular nanomaterial based on Py-modified HMSN and CP5-functionalized ultrasmall CuS nanogates via surface post-modification and supramolecular host-guest strategy for dual-targeted synergistic chemo-photothermal therapy. HMSN-Py ensured the efficient loading and maintained the stability of DOX during drug delivery. CP5-functionalized CuS NPs as nanogates endowed the platform with quadruple-stimuli responsive drug release ability under tumor microenvironments and photothermal performance for PTT, moreover, Fa-mPEG decoration enhanced the active targeting and therapeutic effect. Both *in vitro* and *in vivo* studies indicated that such multifunctional supramolecular platform had good biocompatibility, effective cell internalization, and synergistic chemo-photothermal treatment of tumors. The marriage of HMSN-Py and CP5-functionalized ultrasmall CuS nanovalves with the aid of Fa-mPEG opens a new avenue for the construction of multi-stimuli responsive multifunctional supramolecular drug delivery platform for synergistic chemo-photothermal therapy.

## 4. Experimental Section

**Materials.** CTAB, TEOS, NH_3_·H_2_O (25%-28%), (3-chloropropyl) trimethoxysilan, CuCl_2_·2H_2_O, and Na_2_S·9H_2_O were purchased from Aladdin. DOX and Fa are purchased from Sigma-Aldrich. Calcein-AM, propidium iodide (PI) and Hoechst 33342 were purchased from Tianjin bestbio biotechnology Co., LTD. CP5 was synthesized according to the published procedure 54,57. Ultrapure water with a resistivity of 18.25 MΩ cm at 25 °C was acquired from Milli-Q system.

**Methods.** SEM images and TEM images were recorded on a JEOL JSM 6700F and JEM 2100F instrument with an accelerating voltage of 200 kV. Average hydrodynamic diameter and Zeta potential were measured on a Zetasizer Nano ZS 90 at 25 °C. X-ray photoelectron spectra were recorded on an ESCALAB 250 photoelectron spectrometer. FT-IR spectra and UV-vis-NIR spectra were performed on a Vertex 80 V spectrometer and Shimadzu UV-2550 spectrometer. The fluorescence spectra were recorded on a Shimadzu RF-5301PC fluorescence spectrophotometer. ^1^H NMR spectra was collected at 25 °C on a Bruker AVANCEIII 500 MHz instrument. PXRD results and TGA results were measured on a Rigaku Smart Lab III powder diffractometer and STA 449 thermal gravimetric analyzer in air. Fluorescent images were carried out on an Olympus IX73 instrument. Flow cytometry analysis was obtained on a BD Accuri C6 instrument.

**Preparation of HMSN-Py.** HMSN-Py was prepared according the reported literature with some modifications 58,59. HMSN-OH (100 mg) was dispersed in toluene (10 mL) and sonicated for 20 min, and then the system was stirred at room temperature for 15 min under N_2_ protection. Subsequently, (3-chloropropyl)trimethoxysilan (0.2 mL) and pyridine (1 mL) were quickly injected into the solution. This mixture was stirred at 120 °C for 24 h. HMSN-Py was obtained by centrifugation, washed with ethanol and dried under vacuum for the subsequent experiments.

**Preparation of ultrasmall CP5-CuS 26.** CP5 (28 mg) and CuCl_2_·2H_2_O (16.9 mg) were dissolved in ultrapure water (100 mL) and stirred at room temperature. Then, Na_2_S·9H_2_O (24 mg) was added into the above solution and stirred for 10 min. Afterwards, the mixture solution was heated at 90 °C for 15 minutes and transferred to a dialysis bag with a cut-off of 3500 Da to remove the impurities. The obtained ultrasmall CP5-CuS was stored at 4 °C.

**Preparation of FaPCHD NPs.** DOX (17.4 mg) was added into a solution of HMSN-Py (10 mg) and stirred at room temperature for 24 h. Then, ultrasmall CP5-CuS was added to the solution and stirred for 24 h. Subsequently, excess Fa-mPEG synthesized in the presence of EDC/NHS was added into the above mixture and stirred for another 24 h. The as-prepared FaPCHD NPs was obtained by centrifugation, washed three times with ultrapure water, and dried under vacuum. Similarly, FaPCH NPs was prepared without the addition of DOX. Besides, DOX-loaded mPEG@CP5-CuS@HMSN-Py (PCHD NPs) was prepared without Fa, and mPEG@CP5-CuS@HMSN-Py (PCH NPs) was prepared without Fa and DOX. To obtain the loading capacity of FaPCHD NPs, the supernatant after centrifugation and the washed solution of FaPCHD NPs was collected and measured by UV-vis spectra.

**Photothermal effect of FaPCH NPs.** To evaluate the photothermal effects of the as-synthesized FaPCH NPs, a series of concentrations (0 μg/mL, 100 μg/mL, 200 μg/mL, 400 μg/mL, and 800 μg/mL, respectively) of FaPCH NPs dispersions in aqueous solution (1 mL) were placed in quartz cuvettes and irradiated under 808 nm laser with a power density of 1 W/cm^2^ for 10 min. A digital thermometer was used to record the temperature of the solutions every 10 s, and a thermal camera was used to harvest the thermal pictures at a certain time. Then, 800 μg/mL of FaPCH NPs dispersion was irradiated for 10 min under 808 nm laser with different power densities (0.5 W/cm^2^, 1 W/cm^2^, 1.5 W/cm^2^ and 2 W/cm^2^) to explore the effects of different power densities on the photothermal conversion performance of the FaPCH NPs. Meanwhile, the dispersion was irradiated at 1 W/cm^2^ power density under 808 nm laser and performed five ON/OFF cycles to verify the photothermal stability.

In order to calculate the photothermal conversion efficiency (η), FaPCH NPs dispersion (1 mL) was irradiated with 808 nm laser (1 W/cm^2^) for 10 min. Then the NIR laser was turned off and the solution was cooled down in natural condition. The temperatures of this system at different time were recorded and the photothermal conversion efficiency was calculated according the literature by the following formulas (τ_s_ was calculated by the linear fitting of negative natural logarithm of the temperature increment in the cooling stage to time, T_sur_ and T_max_ is the ambient temperature and maximum temperature of FaPCH NPs dispersion, m_D_ and C_D_ is the mass and thermal capacity of water, T_max,water_ is the maximum temperature of water, I is laser power, and A_808_ is the UV-vis-NIR absorbance of the FaPCH NPs dispersion at 808 nm).


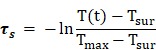



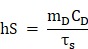



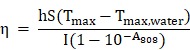


**Controlled drug release experiments.** Owing to the characteristic absorption of DOX in the UV-vis spectra, drug release experiments were performed by monitoring the UV-vis absorption spectra of DOX at the appointed time. Briefly, FaPCHD NPs (1 mg) was packed in a dialysis bag (MWCO 8000) and then immersed in a quartz cuvette containing phosphate buffer solution (PBS, 3 mL) (pH 7.4, 6.0 and 5.0, respectively.) under mild stirring at 37 °C. At predetermined time intervals, the solution was monitored by an UV-vis spectrophotometer. The amount of released DOX was determined by a standard curve made from DOX concentration and absorbance. At the same time, the DOX release experiments were also performed in PBS buffer (pH 7.4) at different temperature (37 °C 45 °C and 60 °C) and in the presence of competitive binding agent (EDA). Meanwhile, in order to investigate the effects of NIR laser on DOX release, the experiment was performed in PBS buffer under 808 nm laser (1 W/cm^2^) ON / OFF treatment.

### Biocompatibility of FaPCH NPs

**MTT assay.** HeLa cells (cancer cell, FR-high expression), A549 cells (cancer cell, FR-low expression), macrophage or IOSE80 cells (normal cell) were seeded in the 96-well plate with a density of 1×10^4^ per well and cultured overnight at 37 °C and 5% CO_2_. various concentrations of FaPCH NPs dispersions were added into the corresponding wells and incubated at 37 °C for 24 h or 48 h. After that, MTT reagent was added to each well and incubated for another 4 h and DMSO (150 μL) was replaced culture medium to dissolve formazan crystals. The absorption was recorded by a microplate reader to calculate cytotoxicity of FaPCH NPs. The equation is as follows (A_e_, A_0_ and A_c_ is the absorbance of the experimental group, control group and blank group, respectively.)


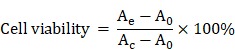


**Hemolysis test.** Red blood cells (RBCs) were separated from the fresh blood and seeded in U-shaped 96-well plate with a density of 7×10^6^ per well (70 μL), then, FaPCH NPs dispersed in PBS buffer (70 μL) were incubated with RBCs under shaking culture at 37 °C for 2 h. For comparison, RBCs treated with equal volume of water and PBS buffer were used as positive and negative control groups, respectively. After centrifugation at 4 °C for 5 min, the absorbance of supernatant was collected and measured by UV-vis spectra at 578 nm. The hemolysis ratio of FaPCH NPs was obtained according to the following equation (A_water_ and A_PBS_ is the absorbance of positive and negative control groups at 578 nm, respectively.):


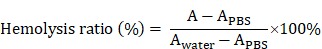


**Photothermal ablation effects and synergistic chemo-photothermal therapy.** HeLa cells were seeded in the 96-well plate with a density of 1×10^4^ per well and cultured overnight. After a part of cells was incubated with FaPCH NPs for 4 h, the cells were irradiated with 808 nm NIR laser (1 W/cm^2^) for 10 min. Then, calcein-AM/PI solution was used to stain the live/dead cells and the results were recorded by fluorescence microscopy. To evaluate the effect of synergistic chemo-photothermal therapy, HeLa cells were treated with various concentrations of free DOX and FaPCHD NPs at same DOX concentrations for 4 h Then, cells were irradiated with 808 nm NIR laser (1 W/cm^2^) for 10 min and incubated for another 20 h. Cell viabilities were measured via MTT assay.

**Intracellular drug release and cellular uptake of FaPCHD NPs.** HeLa cells and A549 cells were seeded in 6-well plates with a density of 3.3×10^5^ per well and cultured overnight. Then, FaPCHD NPs dispersion with a concentration of 33 μg/mL was incubated with cells for different times. After removing the FaPCHD dispersion, Hoechst 33342 was used to stain the cell nuclei, and cells were observed by fluorescence microscopy.

For the cellular uptake, the A549 cells and HeLa cells with a density of 3.0×10^5^ per well were cultured overnight in DMEM medium and 10% fetal bovine serum (the Fa inhibition groups were also added Fa, especially.) and incubated with FaPCHD NPs dispersion at a concentration of 33 μg/mL for different times. After that, the cells were carefully collected. The fluorescence of DOX was recorded by the flow cytometry with 488 nm excitation wavelength.

***In vivo* experiments.** Six-week-old BALB/c female nude mice were subcutaneously injected with 10^6^ HeLa cells after adaptive feeding for one week. After the average tumor volume reached 100 mm^3^, the mice were randomly divided into 7 groups: saline, saline+NIR, FaPCH NPs, FaPCH NPs+NIR, free DOX (5 mg/kg), FaPCHD NPs and FaPCHD NPs+NIR (equivalent to 5 mg/kg free DOX). All mice were injected intravenously at 0, 2 and 4 days. Meanwhile, after 4 h of injection, 808 nm NIR laser (1 W/cm^2^, 5 min) was introduced to saline+NIR, FaPCH NPs+NIR and FaPCHD NPs+NIR groups. Tumor volume and body weight were recorded every two days and the tumor volume was calculated as 0.5×length×(width)^2^. All the mice were sacrificed after 12 days of treatment and the tumor weight was recorded. Meanwhile, the major organs (heart, liver, spleen, lung, and kidney) were collected and used for hematoxylin-eosin (H&E) staining. All the procedures were in accordance with the Guide for the Care and Use of Laboratory Animals approved by wenzhou medical university, China.

**Biodistribution experiments.** Female tumor-bearing BALB/c nude mice were randomly divided into three groups and intravenously injected with free DOX, PCHD NPs (equivalent to 5 mg/kg free DOX), and FaPCHD NPs (equivalent to 5 mg/kg free DOX), respectively. After the injection for 1, 4, and 24 h, the mice were sacrificed. The major tissues (heart, liver, spleen, lung, kidney, and tumor) were washed with PBS and homogenized in acidic alcohol (0.3 N HCl in 50% ethanol), followed by centrifugation at 13000 rpm for 20 min. The obtained supernatant was used to determine the content of DOX by fluorescence spectrophotometry.

## Supplementary Material

Supplementary figures and tables.

## Abbreviations

BET: brunauer-emmett-teller
CP5: carboxylatopillar[5]arene
CP5-CuS: carboxylatepillar[5]arene-functionalized CuS nanoparticles
CTAB: cetyltrimethylammonium bromide
DLS: dynamic light scattering
DOX: doxorubicin
EDA: ethylenediamine
EDS: energy dispersive spectroscopy
EPR effect: permeability and retention effect
Fa: folic acid
FaPCH NPs: Fa-mPEG@CP5-CuS@HMSN-Py nanoparticles
FaPCHD NPs: DOX-loaded Fa-mPEG@CP5-CuS@HMSN-Py nanoparticles
Fa-PEG: folic acid-conjugated polyethylene glycol
FR: folate receptor
FT-IR spectroscopy: Fourier transform infrared spectroscopy
H&E: hematoxylin and eosin
HMSN-Py: pyridinium-modified hollow mesoporous silica nanoparticles
HMSNs: hollow mesoporous silica nanoparticles
NPs: nanoparticles
PBS: phosphate buffer solution
PCHD NPs: DOX-loaded mPEG@CP5-CuS@HMSN-Py NPs
PCH NPs: mPEG@CP5-CuS@HMSN-Py nanoparticles
PEG: polyethylene glycol
PI: propidium iodide
PTT: photothermal therapy
PXRD: power X-ray diffraction
Py: pyridinium
RBC: red blood cell
SEM: scanning electron microscopy
TEM: transmission electron microscopy
TEOS: tetraethylorthosilicate
TGA: thermogravimetric analysis
