# Supplementary material for: Supramolecular nanomaterials based on hollow mesoporous drug carriers and macrocycle-capped CuS nanogates for synergistic chemo-photothermal therapy
Source: Theranostics. 2020 Jan 1;10(2):615–29. doi: 10.7150/thno.40066 (PMC6929989; doi:10.7150/thno.40066)
Supplement: Supplementary file 1 — Supplementary figures and tables. [file thnov10p0615s1.pdf]

Supplementary Material

Supramolecular nanomaterials based on hollow mesoporous drug carriers and macrocycle-capped CuS nanogates for synergistic chemo-photothermal therapy

Jie Yang[1], Dihua Dai[1], Xinyue Lou[1], Lianjun Ma[1], Bailiang Wang[3], Ying-Wei Yang[1,2]

1. International Joint Research Laboratory of Nano-Micro Architecture Chemistry, College of Chemistry, and Department of Endoscopics, China-Japan Union Hospital of Jilin University, Jilin University, Changchun 130012, P. R. China

2. The State Key Laboratory of Refractories and Metallurgy, School of Chemistry and Chemical Engineering, Wuhan University of Science and Technology, Wuhan 430081, P. R. China

3. School of Ophthalmology & Optometry, Eye Hospital, Wenzhou Medical University, Wenzhou, 325027, P. R. China

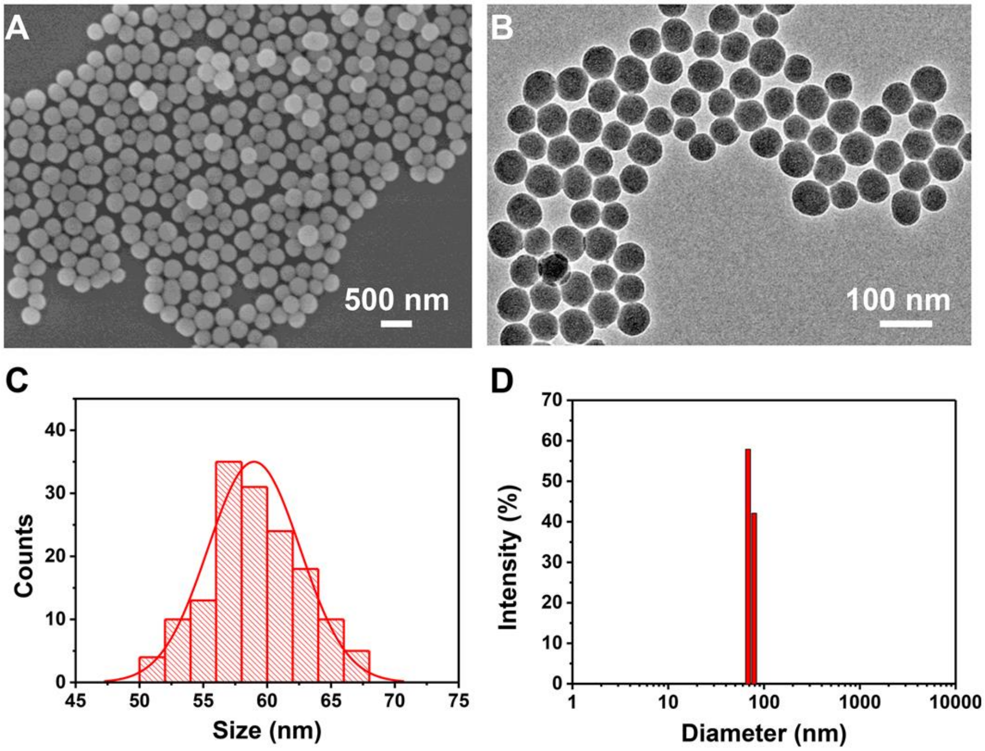

Figure S1. Characterizations of $sSiO_2$ NPs. (A) SEM image of $sSiO_2$ NPs. (B) TEM image of $sSiO_2$ NPs. (C) Size distribution histograms of $sSiO_2$ NPs based on 150 particles of TEM image. (D) Hydrodynamic diameter distribution of $sSiO_2$ NPs by dynamic light scattering.

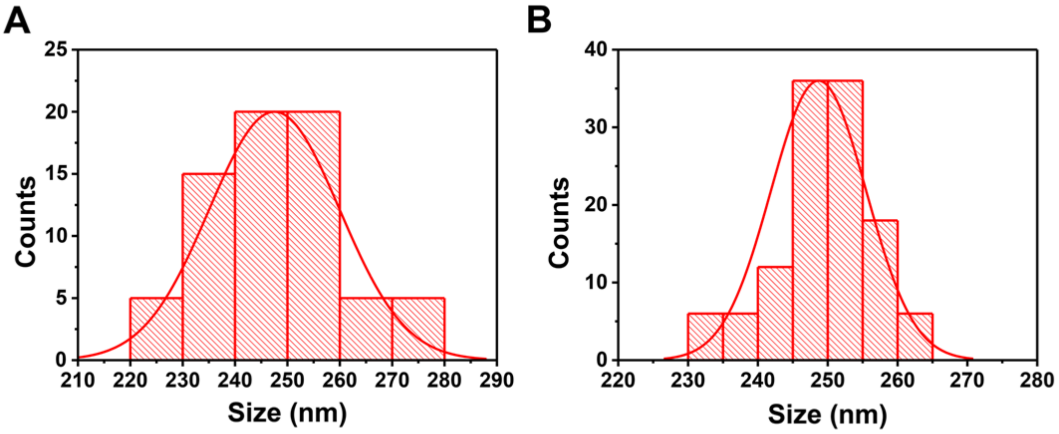

Figure S2. Size distribution histograms of HMSN-Py NPs based on 100 particles of SEM image (A) and 120 particles of TEM image (B).

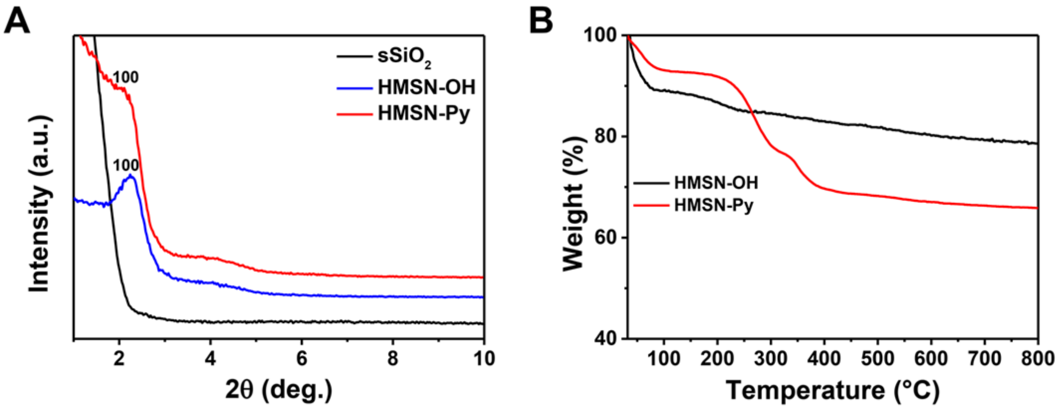

Figure S3. (A) Powder X-ray diffraction (PXRD) spectra of $sSiO_2$, HMSN-OH and HMSN-Py. (B) TGA curves of HMSN-OH and HMSN-Py.

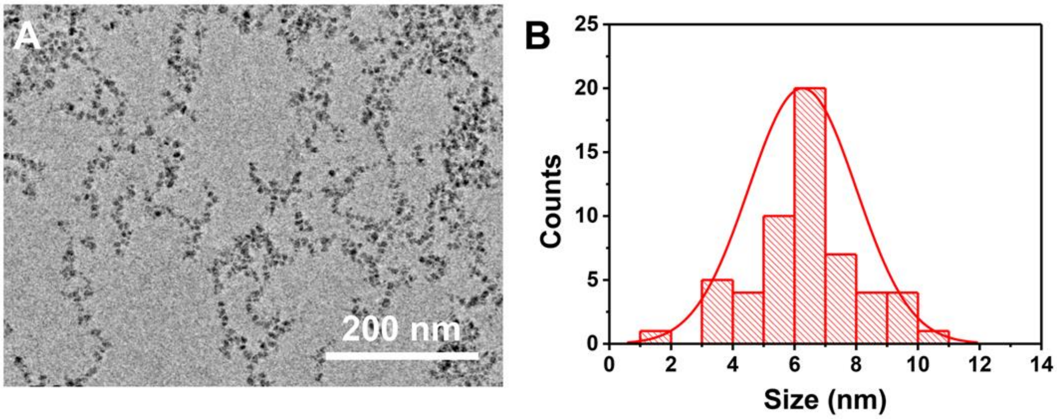

Figure S4. (A) TEM image of CP5-CuS. (B) Size distribution histograms of ultrasmall CP5-CuS based on 56 particles of TEM image.

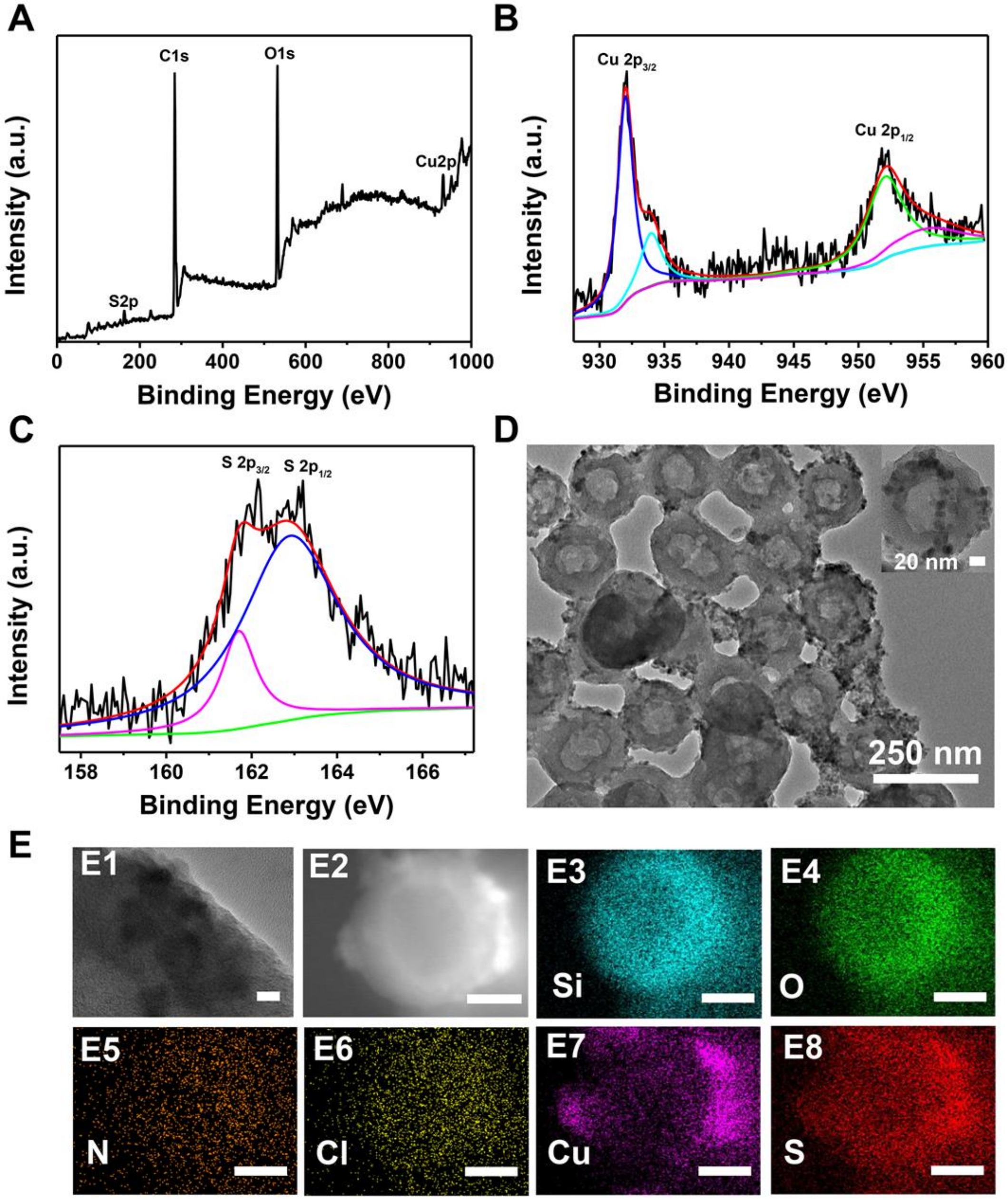

Figure S5. (A) X-ray photoelectron spectra (XPS) of CP5-CuS. XPS spectra of (B) Cu and (C) S in CP5-CuS. (D) TEM image of CuS@HMSN-Py. (E) (E1) High-resolution TEM image of local FaPCH NPs. Scale bar: 10 nm. (E2-E8) Elemental mapping images of FaPCH NPs. Scale bar: 50 nm.

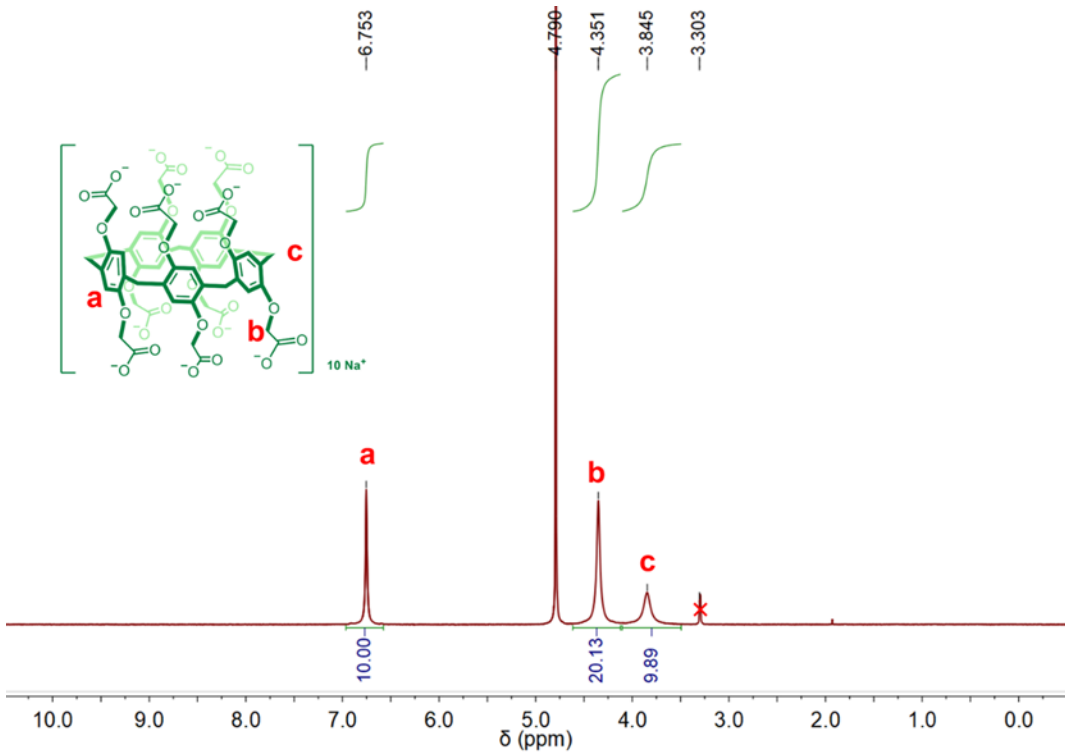

Figure S6. ^{1}H NMR spectrum (500 MHz, 298 K) of CP5 in D_2O. δ 6.75 (s, 10H), 4.35 (s, 20H), 3.85 (s, 10H).

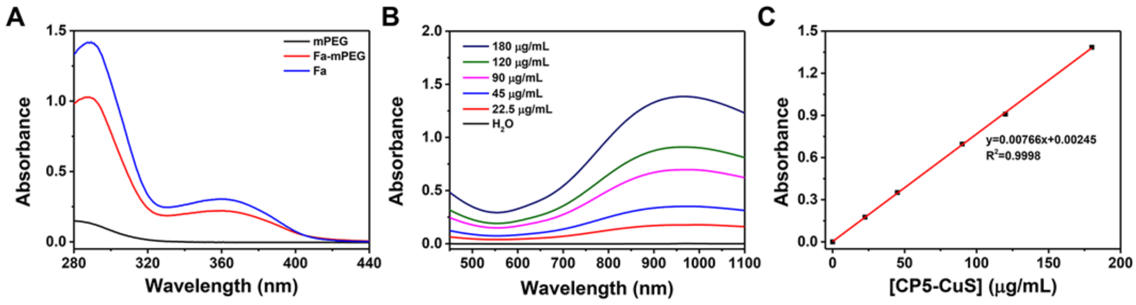

Figure S7. (A) UV-vis absorption spectra of mPEG (black), Fa-mPEG (red) and Fa (blue). (B) UV-vis-NIR absorption spectra of CP5-CuS at different concentrations. (C) The standard curve of CP5-CuS solution at 976 nm.

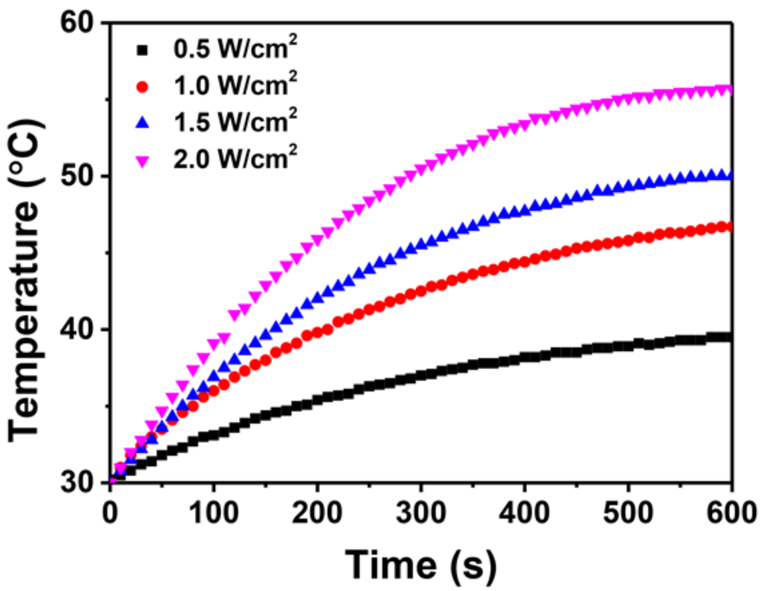

Figure S8. Temperature changes of FaPCH NPs dispersions (800 μg/mL) under 808 nm laser irradiation with different power density of 0.5 W/cm^2, 1 W/cm^2, 1.5 W/cm^2 and 2 W/cm^2 for 10 min.

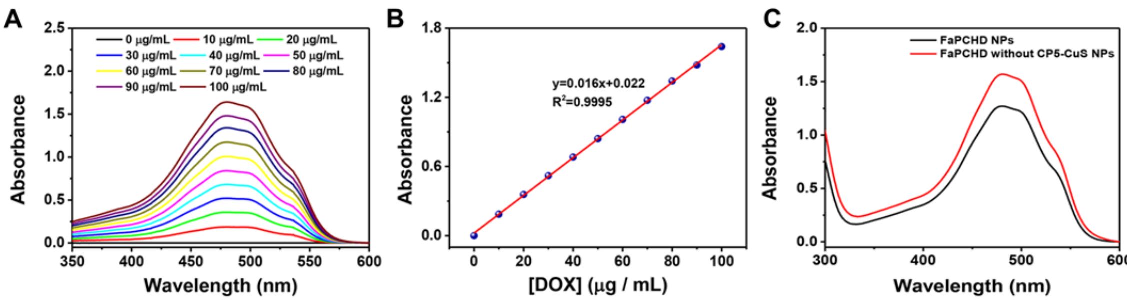

Figure S9. (A) UV-vis absorption spectra of DOX at different concentrations. (B) The standard curve of DOX solution at 480 nm. (C) UV-vis spectra of the supernatant after centrifugation of FaPCHD NPs and FaPCHD without CP5-CuS NPs.

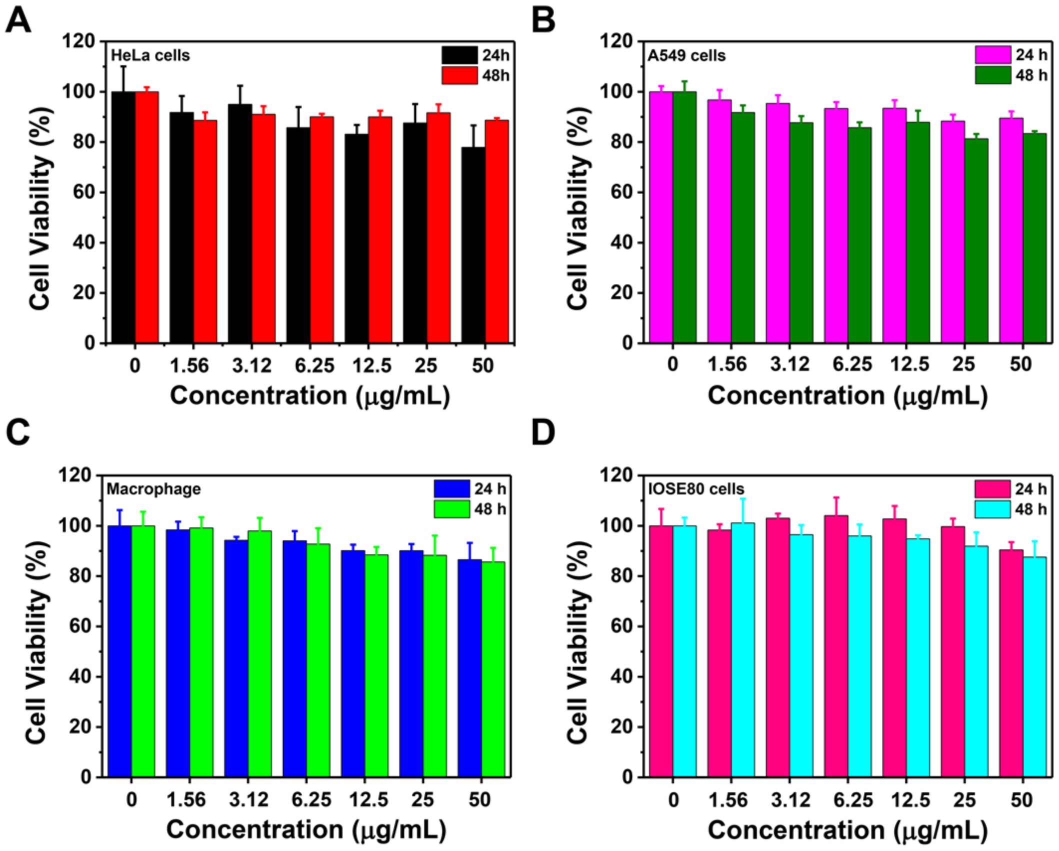

Figure S10. Cell viabilities of (A) HeLa cells, (B) A549 cells, (C) macrophage and (D) IOSE80 cells incubated with various FaPCH NPs for 24 h and 48 h.

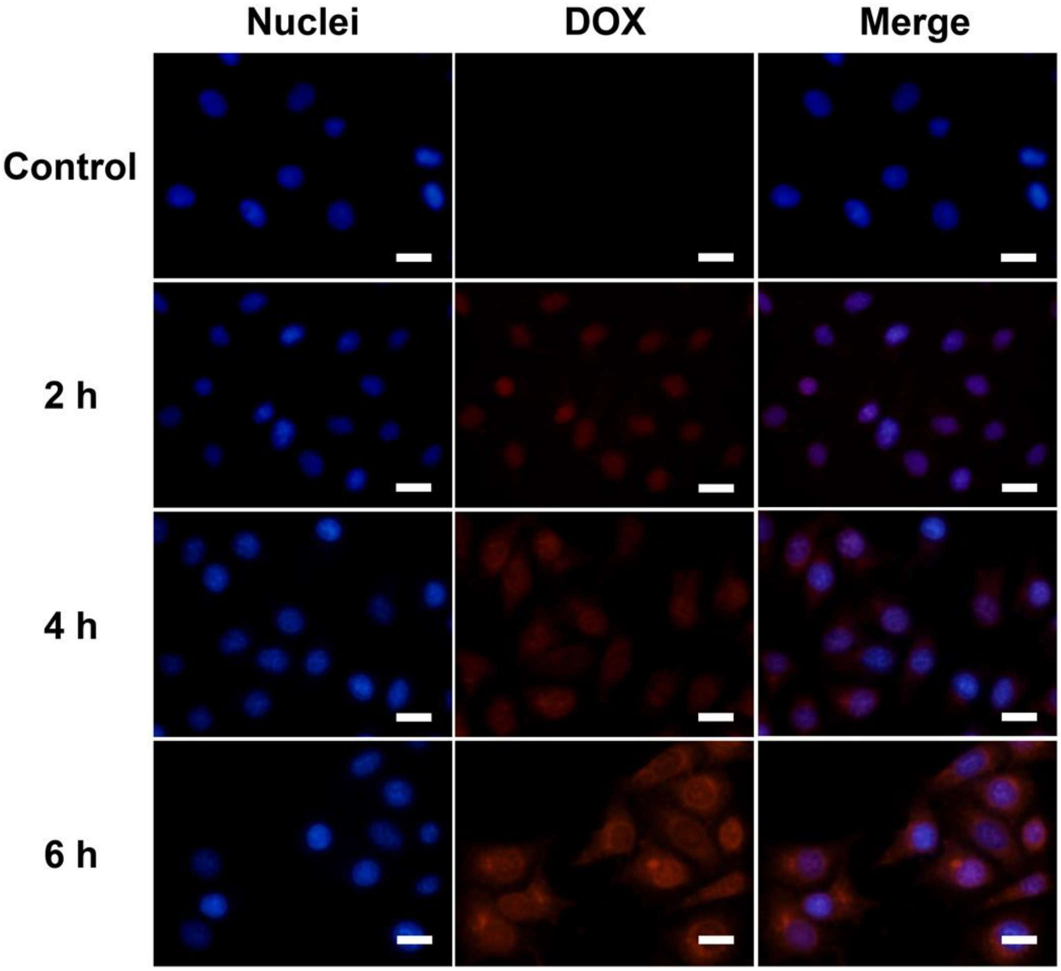

Figure S11. Fluorescence images of A549 cells incubated with FaPCHD NPs for 0 h, 2 h, 4 h and 6 h. The cell nuclei were stained as blue by Hoechst 33342, the red was the fluorescence of DOX. Scale bar: 20 μm.

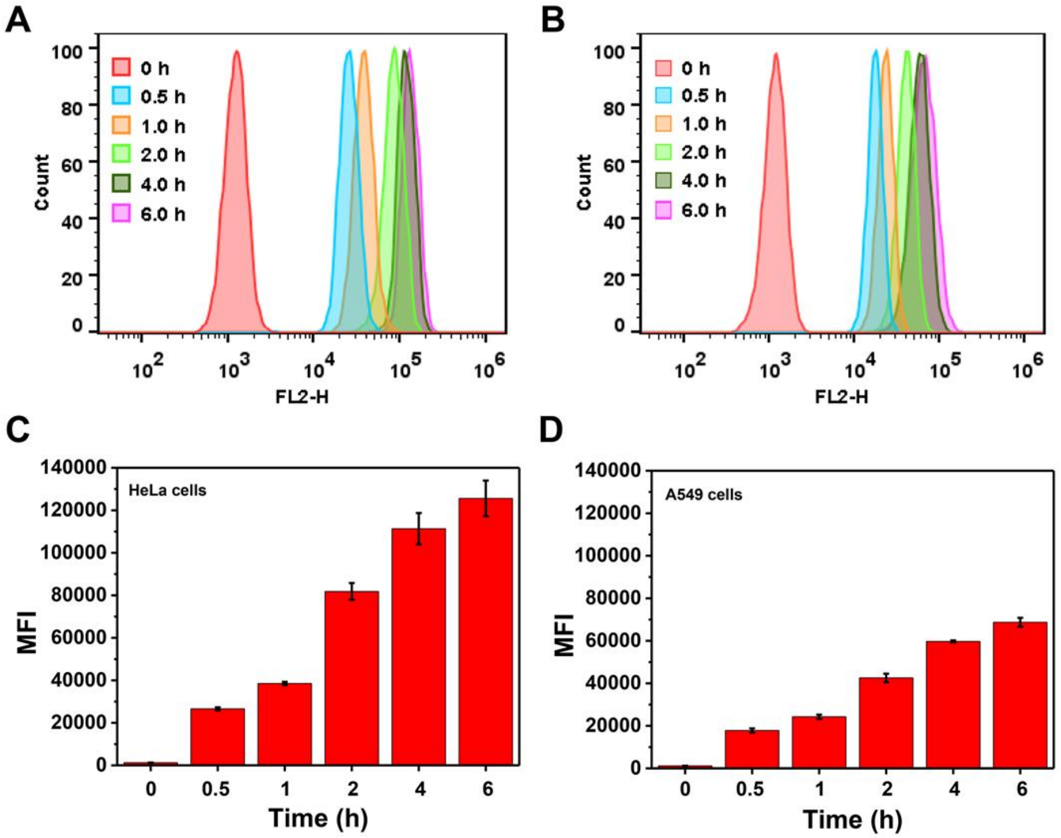

Figure S12. Flow cytometry analysis of (A) HeLa cells and (B) A549 cells incubated with FaPCHD NPs for 0 h, 0.5 h, 1 h, 2 h, 4 h and 6 h. Mean fluorescence intensity of (C) HeLa cells and (D) A549 cells incubated with FaPCHD NPs for various times (0 h, 0.5 h, 1 h, 2 h, 4 h and 6 h).

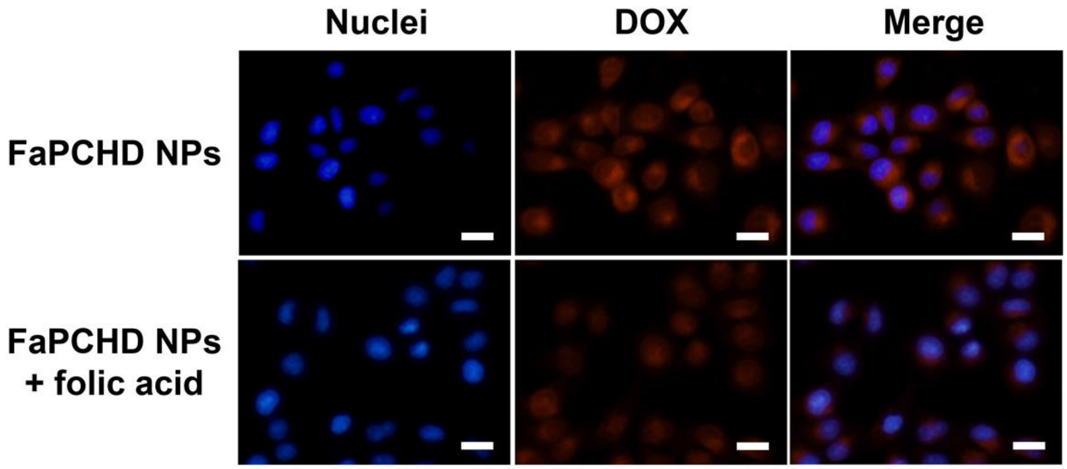

Figure S13. Fluorescence images of HeLa cells incubated with FaPCHD NPs and FaPCHD NPs after pretreatment with free folic acid and FaPCHD NPs for 1 h. The cell nuclei were stained as blue by Hoechst 33342, the red was the fluorescence of DOX. Scale bar: 20 μm.
